# Factors explaining seasonal variation in energy intake: a review

**DOI:** 10.3389/fnut.2023.1192223

**Published:** 2023-07-21

**Authors:** Kyoko Fujihira, Masaki Takahashi, Chunyi Wang, Naoyuki Hayashi

**Affiliations:** ^1^Institute for Liberal Arts, Tokyo Institute of Technology, Tokyo, Japan; ^2^Japan Society for the Promotion of Science, Tokyo, Japan; ^3^Department of Social and Human Sciences, Tokyo Institute of Technology, Tokyo, Japan; ^4^Faculty of Sport Sciences, Waseda University, Saitama, Japan

**Keywords:** season, food intake, appetite, temperature, spring, summer, fall, winter

## Abstract

Maintaining a balance between energy intake and expenditure is crucial for overall health. There are seasonal variations in energy intake, with an increase during spring and winter as well as a decrease during summer. These variations are related to a combination of environmental factors, including changes in temperature and daylight hours; social factors, including events and holidays; and physiological factors, including changes in physical activity and emotions. Accordingly, this review aimed to summarize the environmental, social, and physiological factors that contribute to seasonal variations in energy intake. A review of the current literature revealed that changes in temperature and daylight hours may affect eating behavior by altering homeostatic responses and appetite-related hormones. Additionally, increased participation in events and frequency of eating out, especially during winter vacations, may contribute to increased energy intake. Notably, these findings may not be generalisable to all populations since environmental and social factors can vary significantly depending on the local climatic zones and cultural backgrounds. The findings of the present review indicate that seasonal climate, events, and associated hormonal changes should be taken into account in order to maintain adequate energy intake throughout the year.

## Introduction

Obesity is a global public health concern since it can lead to chronic diseases such as diabetes and cancer (1). According to the World Health Organization, the global obesity rate nearly tripled between 1975 and 2016 (2). Malnutrition, or a lack of sufficient energy intake from the diet among older individuals, is another major health concern. Older adults are at risk of nutritional deficiencies, including anorexia, poor digestion, and nutrient absorption (3). Obesity and malnutrition result from an imbalance between dietary energy intake and expenditure. Therefore, it is important to identify factors that contribute to fluctuations in energy intake from the daily diet in order to maintain a proper energy balance as well as prevent obesity and malnutrition.

Previous meta-analyzes have identified season as a contributing factor to variations in dietary energy intake (4). The pattern of variation in energy intake varies across studies. Meta-analyzes of energy intake during the four seasons (spring, summer, fall, and winter) showed that energy intake was higher in spring than in winter or summer (4). Furthermore, several studies have shown that energy intake is lower in the summer (5–8).

Food availability is often mentioned as a factor influencing seasonal variations in energy intake (4), however, other candidate factors influencing seasonal variations in energy intake include environmental factors such as temperature (9) and daylight hours (5), social factors such as events and holidays (10), and physiological factors such as emotions (6) and physiological activity (11) have not been sufficiently discussed. The influence of environmental, social, and physiological factors on the seasonal variations in energy intake remain unclear.

Determining the factors influencing seasonal changes in energy intake can facilitate the identification of potential contributors to overeating and undernutrition, and thus inform preventive interventions. Therefore, this study aimed to summarize the environmental, social, and physiological factors influencing seasonal variations in energy intake as well as the current literature regarding appropriate energy intake throughout the year.

### Seasonal variations in energy intake

The seasonal variations in energy intake summarized in Table 1 have inconsistent findings. A meta-analysis of studies on seasonal variations in energy intake conducted until 2015 reported that energy intake was greater during spring than during winter and summer; further, it was greater during winter than during summer (4). Other studies conducted after 2015 have reported inconsistent findings regarding seasonal variations in energy intake. A study on individuals aged ≥65 years living in Ankara Province, Turkey, observed greater energy intake during winter than other seasons (6). Specifically, energy intake during winter was higher by ≥557 kcal and ≥ 343 kcal in men and women, respectively than the energy intake during other seasons (6). Another study of adults aged over 45 years in the Ommoord district of Rotterdam, the Netherlands, utilizing a cosinor linear mixed model, revealed that energy intake was the highest in November and the lowest in May (7). In turn, a comprehensive study examining energy intake data from more than 44,000 individuals aged 18–85 years across nine groups in four countries (France, New Zealand, Russia, and Switzerland) revealed no statistically significant variations in energy intake (28). A Spanish study of individuals aged ≥55 observed greater energy intake during spring and fall than during summer (5); moreover, energy intake at lunch was greater during spring than during summer (5). A US study on individuals aged 40–60 years found no significant seasonal variations in energy intake. Taken together, there have been similar but inconsistent findings indicating greater energy intake during winter and spring as well as lower energy intake during summer. This inconsistency could be attributed to the differences in the regional climate and culture of food intake.

**Table 1:** The study locations, energy intake assessment, and energy intake across seasons.

Author	City or Region/Country	Number of participants	Energy intake assessment/Period	Energy intake by each season (kcal)			
				Spring	Summer	Fall	Winter
Aparicio-Ugarriza et al. (5)	Madrid/Spain	28	24 h recall/twice in each season	2,137 ± 152^†^	1,646 ± 108	1,965 ± 121^†^	1,708 ± 89
Arnaud et al. (12)	Havana/Cuba	106	Food record/7 days	1,676 ± 473	1,628 ± 493	1,615 ± 437	1,580 ± 442
Behall et al. (13)	United States	29	Food record/7 days^||^	Males: 2,783 ± 194 Females: 1,863 ± 110	Males: 2,806 ± 209 Females: 1,791 ± 90	Males: 2,779 ± 200 Females: 1,879 ± 87	Males: 2,775 ± 196 Females: 1,791 ± 90
Bernstein et al. (14)	Washington, DC/United States	76	Food record/3–7 days	2,110 ± 627	2,301 ± 642	2,200 ± 581	2,248 ± 638
Capita and Alonso-Calleja (15)	Leo’n /Spain	303	Food records/7 days	-	Males: 2,032 ± 508 Females: 2,080 ± 433	-	Males: 2,913 ± 433^†^ Females: 2,186 ± 536
Ersoy et al. (6)	Ankara/Turkey	31	Food record/3 days	Males: 1,638 ± 435^§^ Females: 1,747 ± 534^§^	Males: 1,655 ± 401 Females: 1,544 ± 379	Males: 1,751 ± 471^†^ Females: 1,827 ± 574^†^	Males: 2,232 ± 504^†^ Females: 1,887 ± 562^†^
Fowke et al. (16)	Shanghai/China	74,958	FFQ	1,692	1,648	1,647	1,665^†^
Fyfe et al. (17)	north-east Scotland/United Kingdom	260	Food records/7 days	Males: 2,225 ± 344 Females: 2,132 ± 234^†‡^	Males: 2,488 ± 176 Females: 1,938 ± 146	Males: 2,505 ± 249 Females: 1,896 ± 167	Males: 2,174 ± 359 Females: 1,942 ± 249^†‡^
Ja Lee et al. (9)	Kentucky/United States	130	Food record/7 days	-	1,450 ± 384	-	1,549 ± 372^†^
Jahns et al. (10)	North Dakota/United States	52	24 h recall/NM	1,900	1,941	1,935	1,940
Ma et al. (18)	Massachusetts/United States	593	24 h recall/3 days	1,942 (SE 23.4)	1,956 (SE 23.5)	1,987 (SE 23.4)	1,958 (SE 22.9)
Mansour et al. (19)	Tehran/Iran	30	Food record/3 days	-	-	-	-
Prasad et al. (20)	Finland	4,880	FFQ	2,723 ± 817	2,723 ± 795	2,771 ± 786	2,723 ± 810
Rao et al. (21)	Pune/India	797	Food record/1 day 24 h recall/1 day	-	1,665 (444–3,755)	-	1,863^†^ (617–3,762)
Rossato et al. (22)	Rio Grande do Sul/Brazil	143	24 h recall/NM	-	-	-	-
Sasaki et al (23)	Iwate, Akita, Nagano, Okinawa/Japan	215	Food record/4–7 days	2,447 ± 457	2,466 ± 4972,350 ± 505 (including Okinawa)	2,491 ± 449	2,415 ± 4132,322 ± 437 (including Okinawa)
Tokudome et al. (24)	Aichi/Japan	80	Food record/7 days	1,811 ± 348	1,792 ± 362	1,852 ± 346	1,825 ± 352
van der Toorn et al. (7)	Rotterdam/Netherland	9,701	FFQ	1,984 (IQR: 1,654–2,391)	1,982 (IQR: 1,625–2,371)	2,021 (IQR: 1,657–2,416)	2,001 (IQR: 1,671–2,396)
Westerterp et al. (25)	Netherland	52	Food records/7 days	-	2,031 ± 286	-	2,246 ± 334
Zhu et al. (26)	Shanghai/China	1,704	Food record/3 days	2,138^†^	1,964	2,084	2,008

Mean ± standard deviation, kJ and MJ have been converted to kcal.

IQR: interquartile range (75% quartile – 25% quartile), SE: standard error, Underlines indicate median values. FFQ: food frequency questionnaire, NM: not mentioned.

* vs Spring, ^†^ vs Summer, ^‡^ vs Fall, ^§^ vs Winter. ^||^ The period of the energy intake assessment is based on previous literature (27).

### Types of seasons

Seasons refer to the several divisions of the year marked by specific changes in weather, temperature, ecology, and daylight hours. The classification of seasons varies across countries and climate zones. In temperate zones, including East Asia and Europe, the year is divided into four seasons (spring, summer, fall, and winter) according to the annual climatic changes. In the tropics or equatorial zones, including Southeast Asia, South America, and parts of Africa, the year is divided into wet and dry seasons since they experience significant changes in the rainfall amount but not in the temperature. There are regions of the world where the weather is relatively stable throughout the year, with no distinct seasons. Such regions include part of tropical areas, for example, Singapore, parts of Hawaii, and the islands of the Caribbean Sea.

Seasonal variations in energy intake have been examined in different areas, including Europe [Spain (5, 15), United Kingdom (17), Netherlands (7, 25), Finland (20), Turkey (6)], North America [United States (9, 10, 13, 14, 18)], Central America [Cuba (12)], South America [Brazil (22)], Asia [China (16, 26), Japan (23, 24), India (21), Iran (19)] (Table 1). Notably, these countries belong to different climate zones with widely varying temperatures and sunlight hours, even during the same season.

## Methods for energy intake assessment

The 24 h recall method (5, 10, 18, 21, 22), food frequency questionnaire (FFQ) (7, 16, 20), and dietary records (6, 9, 12–15, 17, 19, 23–26) have been used to assess seasonal changes in energy intake. The 24-h recall method involves accurately recalling and describing the intake of all food and drink consumed within 24 h before an investigator-led interview (29). Interviews are conducted in person or via telephone or data are collected through online reporting via the Internet. The FFQ estimates the frequency of daily food intake over a time period (30). Specifically, the questionnaire assesses the intake frequency of each food and the amount of each food per intake. The dietary record method involves recording the food and drink consumed by an individual during a specified period (31) using either of the following two methods: the weighing method, in which the weight of food and drink is measured using a scale, and the non-weighing method, in which the approximate amounts of food and drink are recorded.

The dietary survey methods, period, study locations, and energy intake results of previous studies investigating seasonal variation in energy intake are summarized in Table 1. Across these studies, the employed dietary survey methods, including 24-h recall, dietary records, and FFQ, and the number of days for conducting the surveys varied across studies. No definitive relationship can be established between dietary survey methods and seasonal variation in energy intake. Significantly, it is essential to acknowledge that when conducting comparisons of seasonal variations in energy intake among studies, there exists a possibility of encountering potential biases stemming from variations in the utilized dietary survey methods and the period of the survey.

## Factors influencing seasonal variability in energy intake

**Figure 1:**
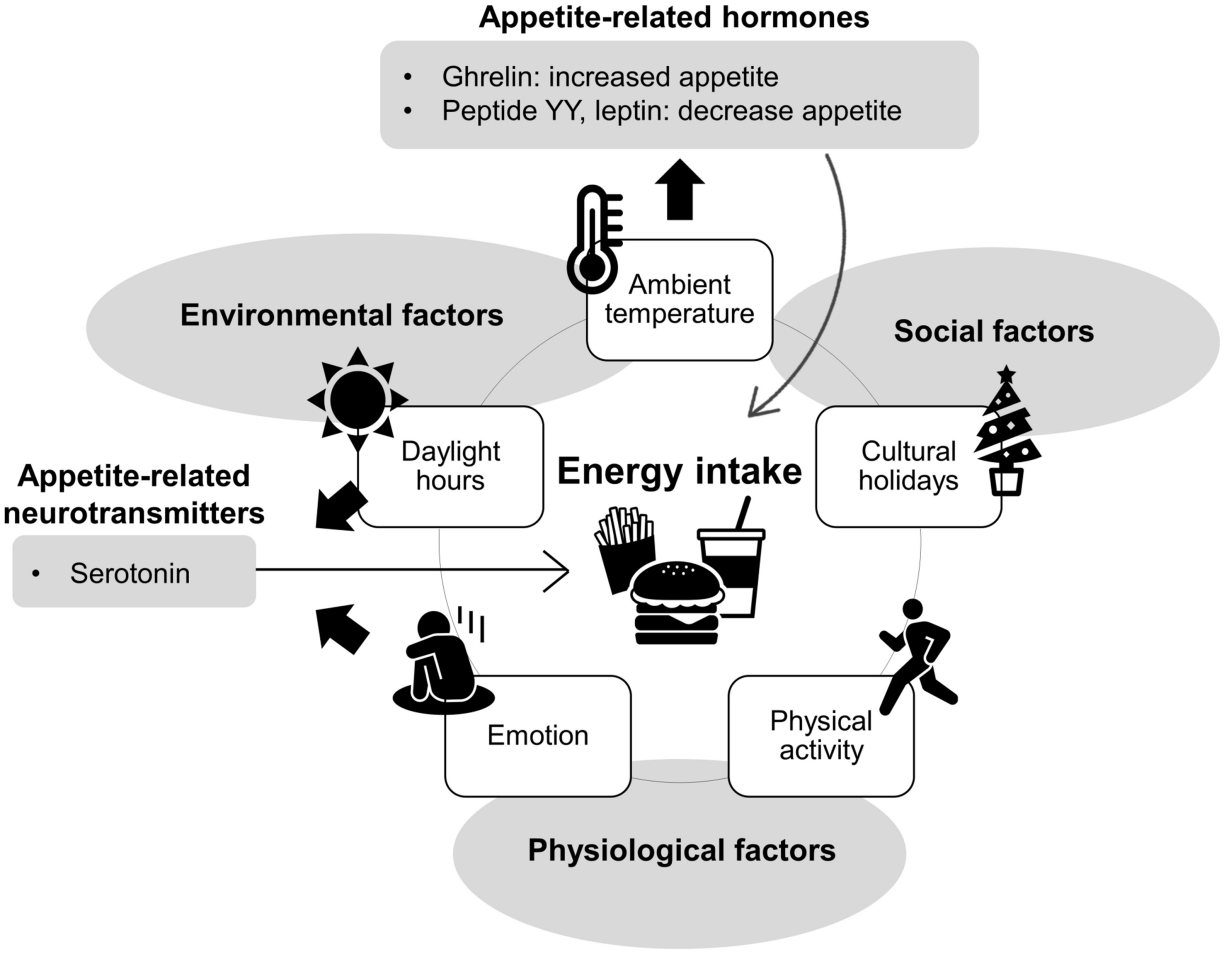
Summarizes the factors influencing seasonal variations in energy intake.

Environmental, social, and physiological factors associated with seasonal variations in energy were shown in Figure 1.

### Food availability

Food availability varies widely by season in some countries, which may impact energy intake. Actually, in areas with pronounced seasonal fluctuations in food availability, individuals tend to consume more food and calories during seasons when food is more abundant (21). This can result in substantial variations in energy intake, particularly in developing and rural areas where food availability is heavily influenced by seasonal changes. For instance, food availability may increase during the post-harvest season, leading to a rise in energy intake, while food depletion may occur during the pre-harvest season, causing a decline in energy intake (21, 32). Conversely, in regions with year-round food availability, such as tropical areas, seasonal variation in energy intake may be less pronounced. Nonetheless, seasonal differences in the types of food available can still affect energy intake, thereby influencing dietary patterns and overall energy intake levels.

### Ambient temperature and energy intake

Ambient temperature, which relates seasonal changes, is known to influence human energy intake (9). A meta-analysis of the effects of ambient temperature on subsequent energy intake at rest and during exercise revealed a small increase and decrease in energy intake under cold and hot conditions, respectively (33). For example, after 5.5 h of thermal exposure at different temperatures (10°C, 20°C, and 30°C), the subsequent energy intake decreased at 30°C compared with that at 10°C and 20°C (34).

The influence of ambient temperature on energy intake could partly involve appetite-related hormones (35–37), which are known to be influenced by ambient temperature. Ambient temperatures are positively correlated with the levels of leptin (35) and peptide YY (36), which are negatively associated with appetite. Cold exposure has been shown to increase ghrelin levels (37), which is positively associated with appetite, and decrease leptin levels (35). A study on seasonal variations in serum leptin levels in young and elderly participants reported that elderly individuals presented higher leptin levels during summer than during spring (38). Variations in the secretion of these appetite-related hormones according to the ambient temperature may contribute to seasonal variations in energy intake.

The variation in temperature across climatic zones and regions, even during the same season, may explain the diverse outcomes observed in the seasonal variations in energy intake across different regions. For example, the mean ambient temperature during summer in Iran is 10°C hotter than that in the Netherlands (39). Therefore, it is important to consider the specific climate of the region when interpreting energy intake patterns during different seasons.

### Daylight hours and energy intake: effect of serotonin

Serotonin, which is a neurotransmitter involved in regulating appetite, may partly contribute to seasonal variations in energy intake. The secretion of serotonin in the brain is closely tied to daylight hours, with serotonin turnover being the lowest during winter (40). On the other hand, the expression of 5-HT1A receptors, which bind serotonin, tend to be higher during summer than during winter in healthy men (41). Moreover, seasonal changes in serotonin secretion may contribute to seasonal variations in energy intake (5). Serotonin regulates feeding and satiety (42). Taken together, seasonal changes in serotonin secretion, which may be influenced by daylight hours, may contribute to seasonal variations in energy intake. Daylight hours are generally longer during summer months and shorter during winter months, which may influence serotonin secretion and energy intake. The number of daylight hours vary greatly according to the latitude of the region. Therefore, it is important to consider the location of the study when analyzing and discussing seasonal variations in energy intake.

### Cultural holidays and energy intake

The fact that the frequency of events encouraging overeating varies across seasons may partially account for the seasonal variation in energy intake. Specifically, certain seasons, like the winter holiday season, often feature a concentration of events involving indulgent meals such as Thanksgiving Day, Christmas, and New Year’s Day, which may result in increased caloric intake. Studies on obese adults have indicated that weight gain can occur due to elevated energy intake during winter vacations (43). Additionally, research suggests that the frequency of eating out increases during the winter holiday season (43), and the quality of healthy eating diminishes (10). These seasonal events and the accompanying episodes of overeating can lead to short-term fluctuations in energy intake.

### Emotions and energy intake

The changing of seasons can affect people’s emotions and appetite. Emotional states vary seasonally (44) and affect eating behavior in humans (45). For example, a variety of factors, such as cold winter weather and reduced exposure to daylight, can lead to depressed or low moods. Negative emotions and appetite have been reported to be associated, specifically young adults have higher energy intake on days when negative emotions are rated higher (45). As a result, some people may turn to food for comfort during the winter months, preferring foods that are high in calories or sugar. Uncontrolled stress alters eating patterns and increases the consumption of highly desirable foods (46). On the other hand, during winter, some individuals may experience a reduced appetite, which could be attributed to seasonal affective disorder-a type of depression that is linked to changes in season and light exposure (47). This may lead to undereating and disinterest in food (47).

### Physical activity and energy intake

There is a complex relationship between seasonal variations in energy expenditure and intake. Energy expenditure is related to an increase or decrease in energy consumption through the homeostatic function. Environmental factors, including temperature, can affect energy expenditure through changes in the metabolic rate and heat production, especially during sleep. For example, energy expenditure is known to vary with ambient temperature (48, 49). Accordingly, compensatory changes in energy intake may occur to maintain energy balance. A previous study reported no significant seasonal changes in energy expenditure (50); contrastingly, a review examining seasonal variations in physical activity and sedentary time found that both total and moderate-to-intense physical activity were higher during summer than during winter (11). Moreover, the sitting time has been reported to be longer during winter than during spring or summer, with people preferring to sit on days with higher precipitation, cooler temperatures, and shorter daylight hours. Although total energy expenditure comprises more than just energy expenditure due to physical activity, seasonal differences in physical activity may result in compensatory changes in energy intake.

## Discussion

Seasonal variations in energy intake were not solely influenced by seasonal food availability, as previously considered. Environmental factors such as temperature and daylight hours can also play a role in influencing the seasonal variability of food intake. Social factors, such as seasonal vacations and events, can also impact energy intake. Moreover, physiological factors such as emotions and physical activity can also affect food choices and portion sizes, resulting in alterations in energy intake. Thus, seasonal energy intake is influenced by a combination of factors, including food availability, temperature, and emotions.

The impact of factors influencing seasonal variations in energy intake may be constrained in specific contexts. While food availability continues to exert a significant influence on energy intake in certain rural areas, studies indicate more limited effects in metropolitan areas. While temperature and daylight hours, which are linked to emotional and physical activity, can influence appetite, the modern environment characterized by controlled indoor temperatures and lighting may attenuate their effects on energy intake. Additionally, it is important to note that the events promoting overeating during specific seasons are contingent upon cultural and religious values.

Thus, the seasonal variability of energy intake is a complex phenomenon that is influenced by a variety of factors, including food availability, environmental factors, social factors, and physiological factors. Understanding these factors is essential to develop effective interventions that can help individuals maintain healthy and balanced diets throughout the year. Regulating daily food intake according to seasonal changes in appetite offers valuable insights into the effective management of obesity and undernutrition through diet. Future studies should incorporate data on climate zones, daylight hours, physical activity, and emotional states to comprehensively examine the factors involved in the seasonal variability of energy intake.

## Author contributions

All authors listed have made a substantial, direct, and intellectual contribution to the work and approved it for publication.

## Funding

This study was supported by the Grant-in-Aid for JSPS Research Fellow under Grant 21J01065.

## Conflict of interest

The authors declare that the research was conducted in the absence of any commercial or financial relationships that could be construed as a potential conflict of interest.

## Publisher’s note

All claims expressed in this article are solely those of the authors and do not necessarily represent those of their affiliated organizations, or those of the publisher, the editors and the reviewers. Any product that may be evaluated in this article, or claim that may be made by its manufacturer, is not guaranteed or endorsed by the publisher.
